# Endoscopic versus open carpal tunnel release for idiopathic carpal tunnel syndrome: a meta-analysis of randomized controlled trials

**DOI:** 10.1186/s13018-014-0148-6

**Published:** 2015-01-28

**Authors:** Dongqing Zuo, Zifei Zhou, Hongsheng Wang, Yuxin Liao, Longpo Zheng, Yingqi Hua, Zhengdong Cai

**Affiliations:** Department of Orthopaedics, Shanghai Tenth People’s Hospital, Tongji University School of Medicine, 200072 Shanghai, China; Department of Orthopaedics, Affiliated People’s First Hospital, Shanghai Jiaotong University, 200080 Shanghai, China

## Abstract

The objective of this study is to do a meta-analysis of the literature and compare the safety and efficacy of endoscopic carpal tunnel release (ECTR) and open carpal tunnel release (OCTR) for idiopathic carpal tunnel syndrome (CTS). A comprehensive literature search of the electronic databases MEDLINE, EMBASE, Google Scholar, and the Cochrane Controlled Trial Register was undertaken for randomized studies reporting carpal tunnel syndrome treated with ECTR or OCTR. The quality of randomized trials was critically assessed. Pooled relative risk (RR) and 95% confidence intervals (CIs) for safety and efficacy outcome variables were calculated by fixed-effect or random-effect methods with RevMan v.5.1 provided by the Cochrane Collaboration. A total of 13 randomized trials were included by total retrieve and riddling. The results of our meta-analysis showed no significant difference in the overall complication rate (RR = 1.34, 95% CI [0.74, 2.43], *P* = 0.34), subjective satisfaction (RR = 1.0, 95% CI [0.93, 1.08], *P* = 0.92), time to return to work (mean difference = −3.52 [−8.15, 1.10], *P* = 0.14), hand grip and pinch strength, and the operative time (mean difference = −1.89, 95% CI [−5.84, 2.06]) between patients in the ECTR and OCTR groups (*P* = 0.16, 0.70, and 0.35, respectively). The rate of hand pain (RR = 0.73, 95% CI [0.53, 0.93], *P* = 0.02) in the ECTR group was significantly lower than that in the OCTR group. ECTR treatment seemed to cause more reversible postoperative nerve injuries as compared with OCTR (RR = 2.38, 95% CI [0.98, 5.77], *P* = 0.05). Although ECTR significantly reduced postoperative hand pain, it increased the possibility of reversible postoperative nerve injury in patients with idiopathic CTS. No statistical difference in the overall complication rate, subjective satisfaction, the time to return to work, postoperative grip and pinch strength, and operative time was observed between the two groups of patients.

## Introduction

Carpal tunnel syndrome (CTS) is one of the most common causes of neuropathy in the upper extremities. It occurs most often in patients aged 30 to 60 years and is two- to threefold more common in women than in men [1]. In many cases, no underlying condition can be diagnosed, rendering it idiopathic, although CTS is associated with rheumatoid arthritis (RA) and other inflammatory arthropathies, trauma, diabetes, acromegaly, hypothyroidism, and pregnancy [2]. The diagnosis mainly depends on clinical symptoms and electrodiagnostic tests. When non-surgical treatments including local steroid injections, splinting, oral steroids, and ultrasound therapy fail, many patients require complete division of the transverse carpal ligament to alleviate their symptoms [3,4].

Since Phalen et al. [5] developed and reported open carpal tunnel release (OCTR) surgery for carpal tunnel syndrome in the 1950s, many researchers have developed and reported the use of short or long incisions limited to the interthenar area of the palm [6,7]. However, the disadvantage of this technique is the possible formation of hypertrophic scars at the thenar and hypothenar eminences accompanied with pain. Okutsu et al. [8] reported the first endoscopic carpal tunnel release (ECTR) in 1987. Since then, the two-portal technique ECTR by Chow et al. [9] and one-portal technique ECTR by Agee et al. [10] have been widely adopted and have become the two standard minimal invasive techniques at present, owing to less pain in the scar area, a better appearance, and a quicker return to work and daily activities, but they are more technically demanding and also require additional equipment as compared with OCTR [11,12]. However, other researchers still prefer OCTR because of fewer technical demands on the OCTR procedure and the lower associated complications and costs [13]. In a systematic review and meta-analysis of randomized trails concerning ECTR and OCTR, Thoma et al. [12] concluded that there was no significant difference between ECTR and OCTR in terms of symptomatic relief. In addition, they found that the results about return to work and hand function were conflicting and that the risk of reversible nerve injury was significantly increased in ECTR patients.

There is no generally accepted consensus for proper surgical management of idiopathic CTS with respect to the efficacy and safety of ECTR and OCTR, especially concerning complications including nerve, vascular, and tendon injuries and wound infections; postoperative hand function; and return to work. The aim of the present meta-analysis was to validate the efficacy and safety of the selection of clinical treatment for such patients. We hypothesize that ECTR may help CTS patients return to work quicker than OCTR; patients with both surgical techniques may present similar postoperative hand function and complication.

## Methods

### Study design

A systematic literature search was performed to identify randomized controlled studies that assessed the efficacy and safety of ECTR and OCTR treatment for idiopathic CTS. The results were systematically analyzed to determine the relationship between the treatment method and the surgical outcome in carpal tunnel syndrome patients.

### Inclusion and exclusion criteria

Studies that reported information pertaining to the efficacy and safety of ECTR and/or OCTR treatment for idiopathic CTS were retrieved, including (1) randomized controlled trials that compared ECTR (any endoscopic technique including Agee’s one-portal and Chow’s two-portal techniques) and OCTR (any open technique, including any type of short incision or long incision limited to the interthenar area of the palm) in idiopathic CTS; (2) studies in which all patients were diagnosed with idopathic CTS; (3) studies that reported follow-ups longer than 4 weeks; and (4) studies that were published in or previously translated into the English language. Studies were excluded if they included patients with arthritis, diabetes, thyroid disease, pregnancy, and any traumatic or operation history of the wrist.

### Database search terms

Electronic searches were performed using the electronic databases provided by Google Scholar [1966 to September 2013], MEDLINE [1966 to September 2013], EMBASE [1974 to September 2013], and the Cochrane Controlled Trial Register [Cochrane Library 2013]. Two independent researchers (Zuo and Wang) conducted literature searches using the search keywords “carpal tunnel release”, “endoscopic”, “open”, “versus or Vs”, and “Randomized or randomization”, with various combinations of the operators “AND”, “NOT”, and “OR”.

### Risk of bias and quality assessment

Eligible studies were evaluated for inclusion by two independent reviewers, and the level of agreement between the reviewers was recorded. Inclusion of resultant titles was determined by manual screening of the titles and abstracts, followed by full-text screening by the same reviewers. Two reviewers independently assessed risk of bias (ROB) of randomized controlled trials (RCTs) and methodological quality of systematic reviews using the 12 validity criteria [14] of the Cochrane Collaboration ROB tool and the revised Jadad scale, respectively.

The Cochrane ROB tool addresses threats to several internal validity domains (selection, performance, detection, attrition, reporting, and other pre-specified bias). The ROB for performance, detection, and attrition was assessed for a priori defined groups of objective and subjective outcomes separately and was classified as high, low, or unclear. Afterwards, for each RCT, within-study summary ROB rating was derived for subjective and objective outcomes. At data synthesis stage (evidence grading), the across-study average summary ROB was determined and assigned to each outcome of interest.

Two reviewers assessed the quality of the studies included independently, and the revised Jadad scale [15] was used to perform the quality assessment. This scale includes the random sequence production (2 points), allocation concealment (2 points), appropriateness of blinding (2 points), and description of dropouts and withdrawals (1 point). The total score is 7 points: 0–3 points mean poor quality, and 4–7 points mean high quality.

### Outcome measurement and definition

Patient outcome measurement included complications, operative time, postoperative subjective satisfaction in at least 12 weeks after surgery, hand pain rate 12 weeks after surgery, hand grip and pinch strength, and the time to return to work. Complications were considered as primary outcome, and other outcomes were secondary. (1) Complications referred to any nerve or muscle tendon injury, hematoma, wound infection, or dehiscence after surgery. (2) Subjective satisfaction referred to patient satisfaction with the surgical outcome at postoperative visits 12 weeks after surgery. (3) Hand pain referred to patient complaint of scar tenderness or pillar pain as assessed by the visual analogue score (VAS) ranging from 0 to 10 points, where a VAS score >3 was analyzed in the study. (4) Hand grip and pinch strength referred to hand grip and pinch strength 12 weeks after surgery as assessed by the equipment and measured in kilograms. (5) Operative time referred to the time from tourniquet inflation to wound suture. (6) Time to return to work referred to the first day after surgery to the time of returning to work.

### Statistical analysis

All data were analyzed using RevMan v.5.1 software (Cochrane Collaboration, Copenhagen, Denmark). Relative risk (RR) and 95% confidence intervals (CIs) were reported. Heterogeneity between studies was assessed using Cochrane’s *Q* test with a *P* value equal to 0.1. The *I*^2^ (variability) statistic is the percentage of total variation across studies due to heterogeneity. A random-effect model was used for heterogeneous data, and sensitivity analysis was conducted to predict the potential source of heterogeneity; otherwise, a fixed-effect model was used. Meta-analysis of pooled relative risk was performed. *P* values less than 0.05 were considered statistically significant (*P* < 0.05).

## Result

### Literature search

**Figure 1:**
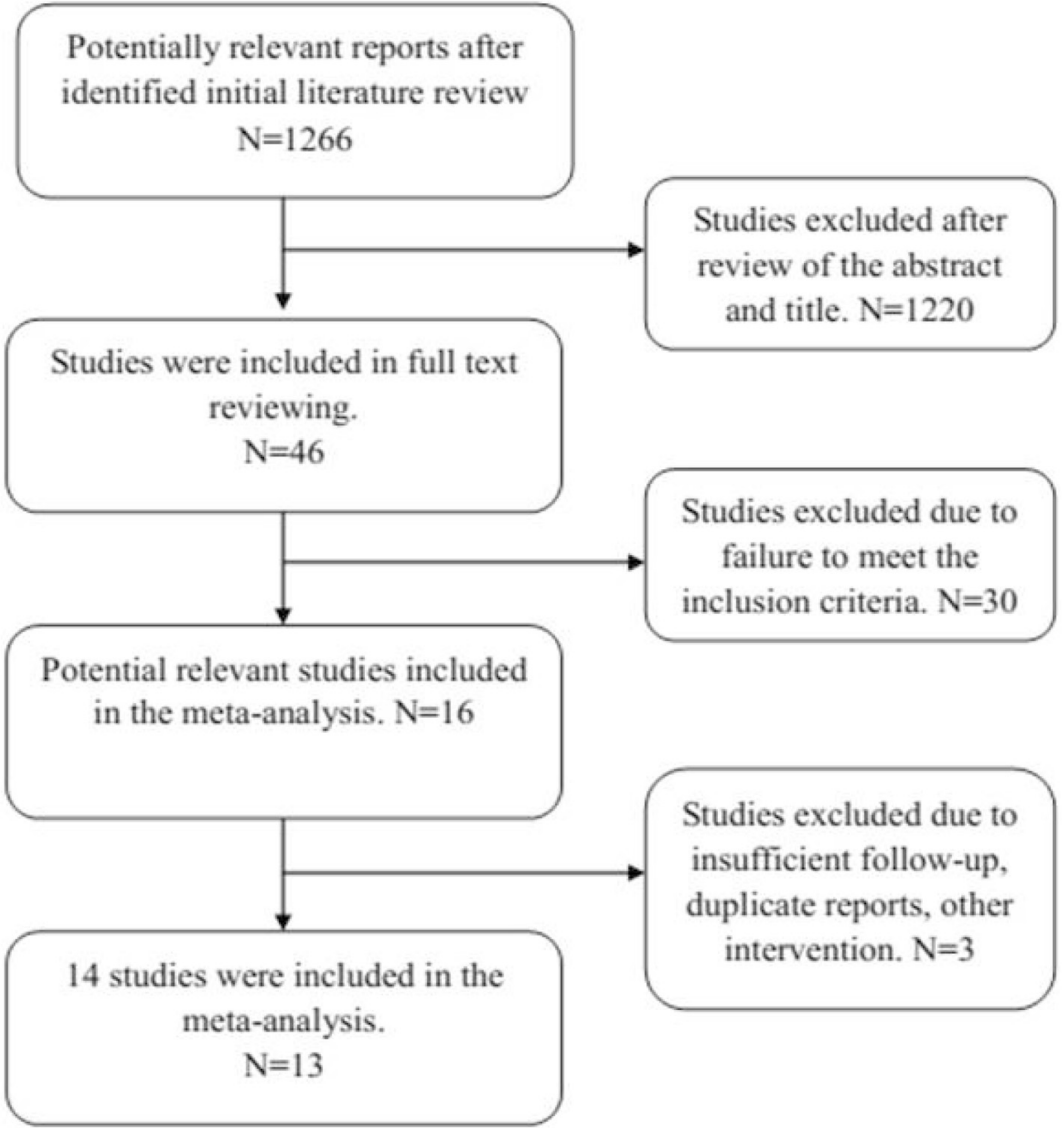
**Study inclusion.**

Initial electronic database searches yielded 1,266 relevant titles, of which 1,250 were excluded due to failure to meet the inclusion criteria. The remaining 16 articles were subjected to full-text review, resulting in exclusion of two additional articles due to failure to meet the inclusion criteria, mostly due to inappropriate comparison methods [16] or insufficient follow-up [17]. One randomized clinical trial by Erdmann et al. [18] reported a total 105 CTSs treated either with ECTR or OCTR; only 43% of patients were diagnosed with idiopathic CTS, and thus, it was excluded. In addition, although 13 randomized studies were included in the meta-analysis conducted by Thoma et al. [12] in 2004, six studies of the meta-analysis were not published in English and were excluded due to the difficulty of obtaining the detailed original information. Study inclusion is detailed in Figure 1. Systematic review and meta-analysis were conducted using the remaining 13 included studies [19-21,6,22-27,10,28,7]. The detailed literature search is shown in Figure 1.

### Study characteristics, risk of bias, and quality assessment

The 13 included randomized controlled studies reported a total of 1,315 hands with idiopathic CTSs treated with either ECTR or other OCTR methodologies. The follow-up period for each study ranged from 1 to 52 months. Publication dates ranged from March 1992 to January 2013. ECTR treatment was administered in 688 hands, and the remaining 627 hands underwent OCTR. Patient demographics, follow-ups, and patient characteristics of the 1,315 hands are listed in Table 1.

**Table 1:** **Study characteristics of randomized studies included in the meta-analysis**

**Study**	**Study design**	**Publication year**	**Country**	**Number (hands)**	**Gender (F/M)**	**Age (year)**	**Treatment**	**Study visits (week postoperative)**	**Efficacy variables**	**Complication**
Agee 1992	Randomized Ten-center study	1992	US	ECTR: 65 OCTR: 82 147 hands in 122 patients	UN	UN	Agee’s one-portal procedure Regional block or general anesthesia	1, 2, 3, 6, 9, 13, 26	Grip strength, pinch strength, Semmes-Weinstein monofilament sensory mapping, Phalen’s wrist flexion test, Tinel’s test, manual motor testing, time return to work	ECTR: 4/65 (2 partial transection, 2 transient ulnar neurapraxia) OCTR: 4/82 (1 deep motor branch of the ulnar nerve, 1 bowstringing of the digital flexor tendons, 2 wound dehiscence)
Brown 1993	Prospective, multicenter, randomized study	1993	US	ECTR: 84 OCTR: 85 169 hands in 151	31/53 23/62	57 55	Two-portal technique	3, 6, 12	Strength, pinch strength, satisfaction, pain	ECTR: 4/84 (1 partial transection, 2 nerve injury, 1 wound hematoma) OCTR: 0/85
Sennwald and Benedetti 1995	Prospective randomized study	1995	Switzerland	ECTR: 25 OCTR: 22	19/6 18/4	48.6 57	One-portal procedure Regional anesthesia	4, 8, 12	Pain, grip, key-pinch strength, and ability to return to work Operative time	ECTR: 1/25 (1 neurapraxia) OCTR: 2/22 (1 RSD, 1 hypotrophic scar)
Dumontier 1995	Prospective randomized study	1995	France	ECTR:56 OCTR:40	49/7 36/4	53.4 50.7	Two-portal technique	2, 4, 12	Numbness, pain, return to work, pinch and grip strength	ECTR: 2/56 OCTR:2/40 (2 reflex sympathetic dystrophy for both groups)
Jacobsen 1996	Prospective randomized study	1996	Sweden	ECTR: 16 OCTR: 16 29 patients	11/5 12/4	UN	Two-portal technique	2, 6, 24	Return to work, patient satisfaction	ECTR: 3/16 (3 transient numbness) OCTR: 0/16
MacDermid 2003	Prospective randomized study	2003	Canada	ECTR: 91 OCTR: 32	62/29 22/10	45 53	Two-portal Chow’s procedure	1, 6, 12	Symptom severity, pain, pinch, grip strength, satisfaction	ECTR: 0/91 OCTR: 0/32
Ferdinand 2002	Prospective randomized blinded study	2002	Scotland	ECTR: 25 OCTR: 25	20/5 20/5	54.88	Two-portal	6, 12, 26, 52	Return to work, day off ADL score, satisfaction, operative time	ECTR: 1/25 (wound pain) OCTR: 3/25 (2 persisting pain, 1 nerve injury)
Trumble 2002	Prospective multicenter randomized study	2002	US	ECTR: 97 OCTR: 95	48/27 47/25	56 56	One-portal	2, 4, 8, 12, 26, 52	Symptom severity score, function score, operative time, satisfaction score, median time return to work, cost	ECTR: 0/97 OCTR: 2/95 (2 reflex sympathetic dystrophy)
Wong 2003	Prospective randomized study	2003	HK	ECTR: 30 OCTR: 30	28/2 28/2	47 47	Two-portal Intravenous regional block	2, 4, 8, 12, 16, 24, 48	Wound and pillar pain, pinch and grip strength, two-point discrimination power, operative time	ECTR: 0/30 OCTR: 0/30
Atroshi 2006	Prospective randomized study	2006	Sweden	ECTR: 63 OCTR: 65	44/19 52/13	44 44	Two-portal technique	3, 6, 12, 48	Pain in scar, median postoperative work absence, severity of symptom, functional score, QOL, hand sensation, operative time	ECTR: 2/63 (2 recurrence of symptoms, 1 for OCTR) OCTR: 1/65
Soichi Ejiri 2012	Prospective randomized controlled study	2012	Japan	ECTR: 51 OCTR: 50	48/3 43/7	59 58	Okutsu’s one-portal technique Local anesthesia	4, 12	Change in subjective symptom, impairment in daily activity, APB-DL, sensation, muscle strength	ECTR: 3/51 (3 exacerbation of symptoms) OCTR: 0/50
Larsen 2013	Prospective Single-blind randomized controlled study	2013	Denmark	ECTR: 30 OCTR: 30	22/8 48/12	54 45	One-portal technique	1, 2, 3, 6, 12, 24	Pain VAS score, paresthesia, grip strength, range of motion, pillar pain, duration of sick leave	ECTR: 2/302 (neurapraxia) OCTR: 2/60 (2 infection)
Ho Jung Kang 2013	Prospective randomized controlled study	2013	South Korea	ECTR: 52 OCTR: 52	48/4 48/4	55 55	One-portal technique General anesthesia	12	BCTQ-S, BCTQ-F, DASH, intraoperative tourniquet time, pain, scar or pillar pain	UN

*UN* unknown, *BCTQ-S* Boston Carpal Tunnel Questionnaire score, *DASH* Disabilities of the Arm, Shoulder and Hand, *APB-DL* abductor pollicis brevis-distal latency, *ADL* activity of daily living.

**Figure 2:**
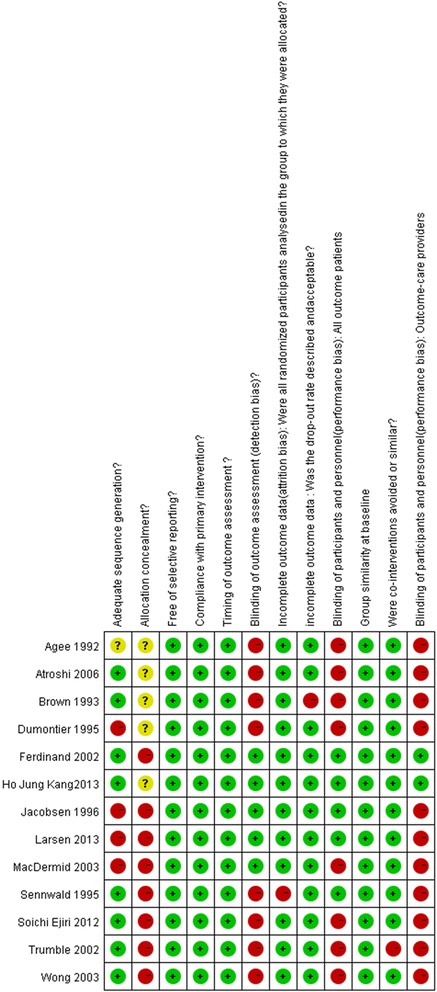
**ROB assessment for included RCTs.**

Overall, eight RCTs reported an adequate method for random sequence generation (low ROB). Only five RCTs (5/13) had lower risks of detection bias for outcomes. Most RCTs failed to report the blinding status of patients, study personnel, and/or outcome assessors. Attrition bias was judged at low risk for at least 11 RCTs. All RCTs were at low risk of selective reporting of outcome. See the ROB assessment for the included RCTs (Figure 2).

All the 13 clinical trials were level-II evidence. For the 12 ROB criteria by Fulan 2009, studies included in the current meta-analysis presented a high evidence level (at least six criteria for each study). For the revised Jadad scale, only two studies had 3 points, indicating a relatively poor quality, and the other studies had 4–7 points, indicating a high quality. The detailed Jadad score of 14 studies is shown in Table 2.

**Table 2:** **Study quality score by modified Jadad score**

**Study included**	**Study design**	**Randomization**	**Blind method**	**Allocation concealment**	**Withdraw and dropout**	**Revised Jadad score**	**Level of evidence**
Agee 1992	RCT	1	1	0	1	3	II
Brown 1993	RCT	2	1	0	1	4	II
Sennwald and Benedetti 1995	RCT	2	1	0	1	4	II
Dumontier 1995	RCT	2	1	0	1	4	II
Jacobsen 1996	RCT	1	1	0	1	3	II
MacDermid 2003	RCT	2	1	0	1	4	II
Ferdinand 2002	RCT	2	1	0	1	4	II
Trumble 2002	RCT	2	2	0	0	4	II
Wong 2003	RCT	2	2	0	1	5	II
Atroshi 2006	RCT	2	1	0	1	4	II
Soichi Ejiri 2012	RCT	2	2	0	0	4	II
Larsen 2013	RCT	2	2	0	1	4	II
Ho Jung Kang 2013	RCT	2	2	1	1	5	II

### Heterogeneity of studies

The variability (*I*^2^) in the results of the six studies used to compare operative time between ECTR and OCTR patients demonstrated a true difference in the treatment effect of 97%, indicating heterogeneity; therefore, the random-effect model was used to adjust for comparison of heterogeneity. The study by Sennwald [25] was excluded by sensitivity analysis in the current analysis. The studies included in evaluating patient hand pinch [19,20] also indicated a relatively high heterogeneity (with *I*^2^ of 87%). The *I*^2^ value in three studies [20,22,24] used to compare subjective satisfaction between ECTR and OCTR patients was 21%, indicating a relatively low heterogeneity. In evaluation of postoperative pain rate, the *I*^2^ value of four included studies was 44%, indicating a heterogeneity; sensitivity analysis was employed and found the study of Dumontier et al. [21] to contribute to the heterogeneity (when the study of Dumontier 1995 was eliminated, the heterogeneity *I*^2^ value was 0%). The heterogeneity *Q* test in comparing patient hand grip strength, time to return to work, reversible nerve injury, and the overall complication rate exhibited no heterogeneity, and therefore, the random-effect model was used.

### Primary outcome: complications

Complete data for the complication rate were available in all included studies, allowing for use of all 13 studies in the analysis of the overall complication rate. Postoperative nerve injury was mentioned in eight studies [10,20,21,28,25,26,22,7]. Pooled data indicate that ECTR patients had a higher nerve injury rate as compared with OCTR patients (RR = 2.38, 95% CI [0.98, 5.77], test for overall effect: *Z* = 1.92 (*P* = 0.05)).

**Figure 3:**
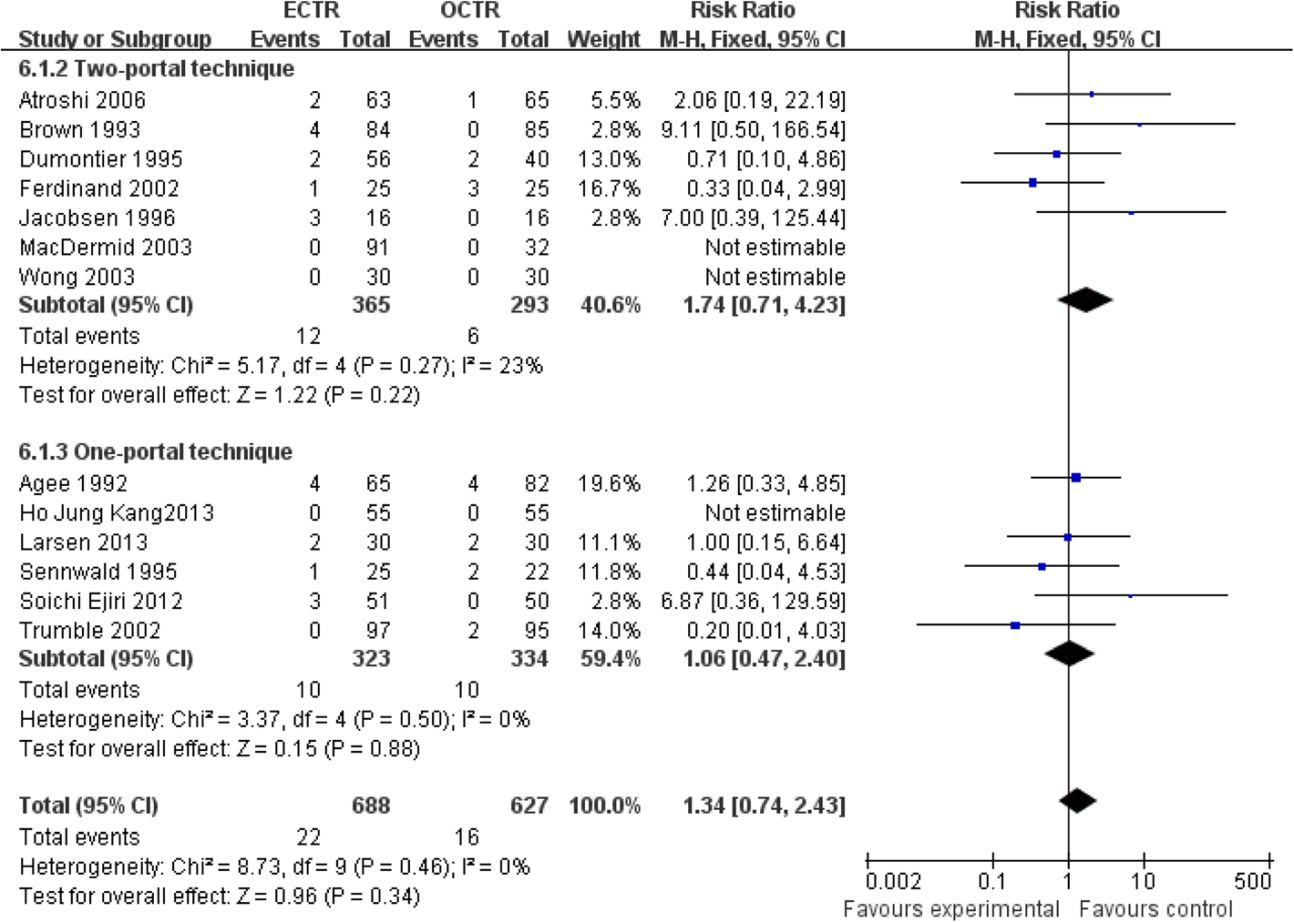
**Forest plot of overall complication and subgroup analysis.**

The overall complication rate did not differ significantly between the ECTR and OCTR groups either in the overall or subgroup related to the comparison of endoscopic technique. The ECTR patients exhibited no significant difference in complication rate (RR = 1.34, 95% CI [0.74, 2.43], and overall effect: *Z* = 0.96; *P* = 0.34) as compared with OCTR patients. Noticeably, the complication risk was higher in two-portal ECTR patients than in one-portal ECTR patients (RR = 1.74, 95% CI [0.71, 4.23] versus RR = 1.06, 95% CI [0.47, 2.40]). The forest plot of overall complication and subgroup analysis is shown in Figure 3.

### Operative time

**Figure 4:**
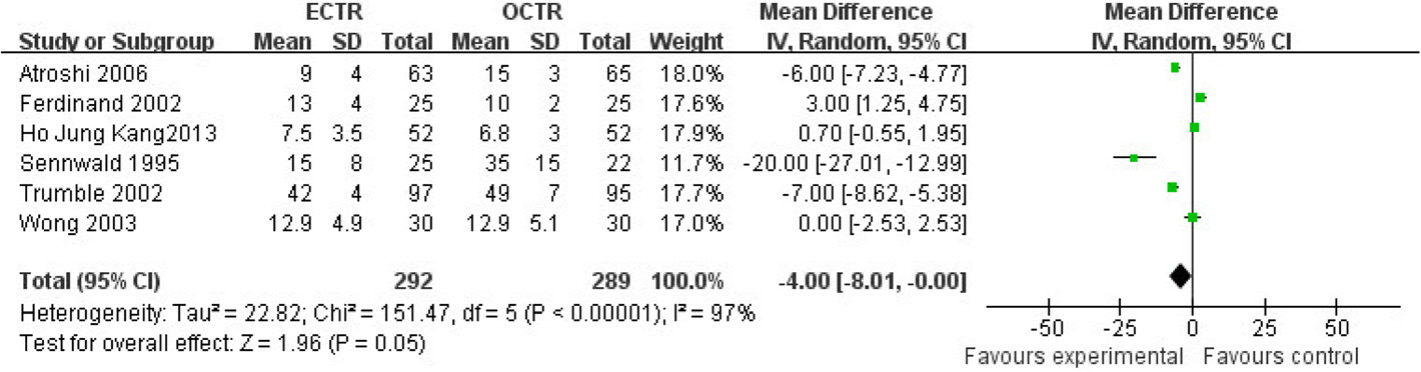
**Forest plot showing comparison of operative time of five studies.**

Six studies [19,22,23,25-27] evaluated the difference in operative time between the ECTR and OCTR groups. Most researchers [19,25-27] reported a longer time demand in OCTR surgery. In contrast, Ferdinand et al. [22] and Kang et al. [23] reported that the ECTR procedure needed a longer time as compared with OCTR surgery. Sensitivity analysis indicated that the study of Sennwald [25] was heterogeneous and thus was excluded from the operative time analysis. The pooled data of five studies indicated that ECTR did not significantly reduce the operative time as compared with OCTR (mean difference = −1.89, 95% CI [−5.84, 2.06], test for overall effect: *Z* =0.94; *P* = 0.35). The forest plot showing the comparison of operative time of five studies is shown in Figure 4.

### Patient subjective satisfaction

**Figure 5:**
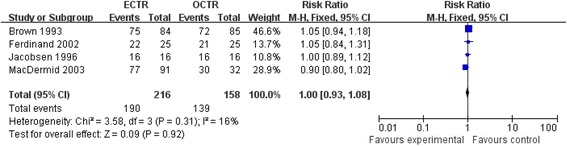
**Patient subjective satisfaction.**

Four studies [20,22,28,24] provided complete data regarding patient subjective satisfaction, indicating that patients in the ECTR group had no significant difference in improvement in subjective satisfaction (RR = 1.0, 95% CI [0.93, 1.08], test for overall effect: *Z* = 0.09 (*P* = 0.92)), as compared with patients in the OCTR group. Detailed information is shown in Figure 5.

### Hand grip and pinch strength

**Figure 6:**
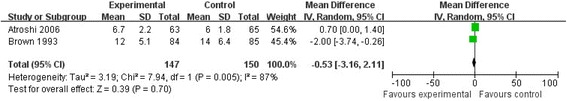
**Meta-analysis of hand function.**

**Figure 7:**
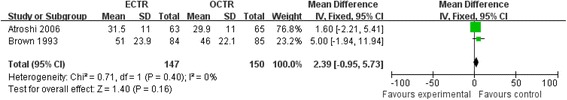
**Comparison of hand function 12 weeks after surgery.**

Two studies [19,20], including 147 ECTR hands and 150 OCTR hands, compared hand grip and pinch strength 12 weeks after surgery. Data of the meta-analysis are shown in Figure 6. The pooled mean difference 12 weeks after surgery was 2.39 (95% CI [−0.93, 5.73], *P* = 0.16) for grip strength and −0.53 (95% CI [−3.16, 2.11], *P* = 0.70) for pinch strength, indicating that the two outcomes did not favor the carpal tunnel release technique specifically. Comparison of hand function 12 weeks after surgery is shown in Figures 6 and 7.

### Pain rate

**Figure 8:**
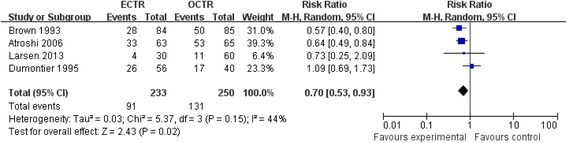
**Comparison of postoperative pain complaints.**

Four studies [19,21,20,7], including 233 ECTR hands and 250 OCTR hands, reported data available for the incidence of hand scar tenderness or pillar pain in ECTR and OCTR patients 12 weeks after surgery. ECTR patients reported lower scar tenderness or pillar pain in the affected wrist (RR = 0.73, 95% CI [0.53, 0.93], test for overall effect: *Z* = 2.43 (*P* = 0.02)) as compared with OCTR patients. Sensitivity analysis was performed by eliminating the study of Dumontier et al. [21] in the current meta-analysis; heterogeneity of the analysis was reduced to 0, but the result showed no significant difference in the pooled data. Comparison of postoperative pain complaint is shown in Figure 8.

### Time to return to work

**Figure 9:**
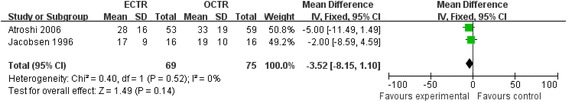
**Comparison of time to return to work.**

Two studies [19,28] evaluated the time for CTS patients to return to work, showing no significant difference between ECTR and OCTR patients (mean difference = −3.52 [−8.15, 1.10], test for overall effect: *Z* = 1.49 (*P* = 0.14)). Comparison of time to return to work is shown in Figure 9. The summary of all outcome variables is shown in Table 3.

**Table 3:** **Summary estimates of outcome variables in the current study**

**Outcomes**	**Number of studies**	**Heterogeneity** ***I*** ^**2a**^ **(%)**	**Pooled relative risk** ^**b**^	**Mean difference**	**95% confidence interval**
Operative time	5	97	NA	−1.89	[−5.84, 2.06]
Patient’s satisfaction	4	21	1.0	NA	[0.93, 1.08]
Grip strength	2	0	NA	2.39	[−0.95, 5.73]
Pinch strength	2	87	NA	−0.53	[−3.16, 2.11]
Return to work	2	0	NA	−3.52	[−8.15, 1.10]
Complication	13	0	1.34	NA	[0.74, 2.43]
Two-portal technique	7	23	1.74	NA	[0.71, 4.23]
One-portal technique	6	0	1.06	NA	[0.47, 2.40]
Nerve injury	8	0	2.38	NA	[0.98, 5.77]

*ECTR* endoscopic carpal tunnel release, *OCTR* open carpal tunnel release, *NA* not applicable.

^a^Heterogeneity test: *I* ^2^ > 50%, random-effect analysis model; *I* ^2^ < 50%, fixed-effect analysis model.

^b^If odds ratio/mean difference >1, favors ECTR; if odds ratio/mean difference <1, favors OCTR.

## Discussion

To provide an accurate and contemporary analysis on carpal tunnel syndrome treatment, the current meta-analysis reviewed 13 methodologically sound randomized controlled studies encompassing 1,315 idiopathic CTS hands treated with OCTR or ECTR. The results clearly indicated that ECTR significantly increased the risk of reversible postoperative nerve injury as compared with OCTR, while the operative time, postoperative overall complication incidence, and hand function were not significantly different between the two groups of patients 3 months after surgery. Despite extensive recent investigation of carpal tunnel release and different surgical procedures for CTS patients, no consensus for proper treatment has been widely accepted. Thus, the current study provides a novel and compelling evidence for contemporary clinical practice for idiopathic CTS patients.

Many efforts have been made to prove the advantages of endoscopic release over open carpal tunnel release treatment option for idiopathic CTS [19,29-33,22,6,11,24,17,34,12,4,27]. As the ECTR technique requires the endoscope to pass through the carpal tunnel as a tight compartment, it inevitably aroused the concern about whether it would cause trauma to the median nerve. Since Okutsu et al. [35] first reported the application of ECTR to CTS treatment in 1989, several studies [20,36,37] have reported ECTR-related postoperative complications. As the carpal tunnel is a cylindrical inelastic cavity connecting the volar forearm with the palm and is bounded by the transverse arch of the carpal bones dorsally, complications including intraoperative injury to the flexor tendons, median ulnar and digital nerves, and superficial palmar arterial arch have been reported when performing the endoscopic procedure. Brown et al. [20] reported a higher risk in CTS patients receiving ECTR treatment and therefore advocated establishment of effective training programs for surgeons, including hand-on courses and cadaveric maneuvers. A systematic meta-analysis of 13 randomized controlled trials by Thoma et al. [12] reported that the risk of causing reversible nerve injury with ECTR was three times as high as that with OCTR treatment; overall postoperative complication risk was not mentioned and calculated in the study. However, the pooled data in the current meta-analysis clearly indicated that patients with ECTR exhibited no significant difference in complication risk as compared with OCTR (RR = 1.34, 95% CI [0.74, 2.43], *P* = 0.34); the conclusion about nerve injury risk in the current study was similar to that in Thoma et al.’s study. The subgroup analysis regarding different endoscopic techniques (one- and two-portal techniques) was consistent with the overall complication risk between ECTR and OCTR, although patients who underwent the two-portal endoscopic technique displayed a slightly higher susceptibility to complication risk (RR = 1.74, 95% CI [0.71, 4.23]).

The evidence from this meta-analysis supports the conclusion that ECTR reduced the incidence of hand pain, while no significant difference was found in operative time as indicated by the follow-up visits 12 weeks after surgery. In the current study, only six studies [19,22,27,25,23,26] reported the time for surgery and three studies [21,20,19] evaluated the incidence of hand pain, showing that ECTR surgery required a similar time (mean difference = −1.89 95% CI [−5.84, 2.06], *P* = 0.35) as compared with OCTR patients. However, it is worth mentioning that the setup of ECTR instruments requires additional time after anesthesia and the setup time was not described qualitatively, thus possibly contributing to the uncertainty of this analysis. Subgroup analysis was not conducted owing to the relatively small sample. The recent conflicting evidence is mainly attributable to methodological discrepancies and different measurement methods between studies. Regarding pain relief, ECTR patients reported significantly less pain during the follow-up interview 12 weeks after surgery (*P* < 0.0001) compared with OCTR patients. Several studies employed VAS (0–10 points) to quantify the pain complaint of patients, thus minimizing the subjective influence on the assessment. But only few data related to the meta-analysis can be retrieved from the published results. In the study by Atroshi et al. [19], the differences in hand pain between 3, 6, and 12 weeks generally became smaller. The changes from 3 weeks to the following follow-up times did not differ significantly between the two groups. The advantage in operative time and hand pain with ECTR could be attributable to the non-use of the Esmarch tourniquet and postoperative splint immobilization. Local infiltration and less invasiveness could also be possible reasons for the current conclusion.

Better hand function recovery promotes early return to work in patients receiving ECTR or OCTR. In a randomized trial by Brown et al. [20] who evaluated hand function and time to return to work in 84 ECTR and 85 OCTR surgeries involving 151 patients with carpal tunnel syndrome, the median time for patients in the ECTR group to return to work was 14 days versus 28 days in the OCTR group (*P* = 0.05). Trumble et al. [26] reported significant advantages of ECTR over OCTR in terms of the time to return to work (18 days versus 38 days, *P* = 0.0086). Pooled data in the current meta-analysis reach agreement with the findings of Brown et al. [12] and Trumble et al. [13] (*P* = 0.14), who reported valid grip and pinch strength 12 weeks after surgery. There was no statistically significant difference in hand grip and pinch strength between the ECTR and OCTR groups. Several studies [19,20,26] found that the hand function was improved in a shorter postoperative time in ECTR patients, while no significant difference was observed in hand grip and pinch function recovery 12 weeks after surgery in the two groups. On the contrary, Ferdinand et al. [22] reported that the endoscopic technique had no significant advantage over OCTR at all stages of postoperative assessment in terms of recovery of muscle strength, hand function, and grip strength. Although similar results were achieved in the current study, the data in three randomized controlled studies could not be combined to determine whether it is also true of large samples and long-term visits. However, the association between the surgical approaches and the recovery of hand function needs to be validated in a further study due to the study quality and the limited sample size in the present meta-analysis. Additional multicenter studies with sufficient and comprehensive data regarding surgical techniques and outcome measure characteristics are required to better evaluate new and improved treatments.

The published meta-analysis by Thoma et al. [38], Vasiliadis et al. [39], and Chen et al. [40] reported a larger sample size and more randomized controlled trials than the current study, but they investigated CTS patients without exclusion of patients with rheumatoid arthritis (RA) and other inflammatory arthropathies, trauma, diabetes, acromegaly, hypothyroidism, and pregnancy; thus, the current study specifically provided a more accurate evidence for idiopathic CTS concerning surgical option with OCTR and ECTR.

### Limitations

This study had several limitations. First, as the overall complication rate including nerve, vascular, and tendon damage and wound infection was calculated with pooled data, and subgroup analysis was only concerned with nerve injury and ECTR technique, we were unable to conduct other specific complication rates of the two surgical techniques. Second, subgroup analysis was not performed regarding the variety of ECTR (one-portal, two-portal, and Okutsu techniques) and OCTR (short incision and long incision limited to the interthenar area of the palm) techniques in outcome variables except for complications, mainly due to the insufficient sample size and limited available data. Third, studies included in the current meta-analysis by Ferdinand et al. [22], Wong et al. [27], and Kang et al. [23] consisted of patients with bilateral carpal tunnel syndrome. Some outcome variables such as patient subjective satisfaction, time to return to daily work, and postoperative pain of one hand in the same patients could be influenced by the other hand that underwent a different surgical technique, thus adding to another confounder for the current study and making the study less convincing.

## Conclusion

In summary, the current study included the pooled data from, to the best of our knowledge, the largest study sample involving 1,315 hands of patients having idiopathic carpal tunnel syndrome in 13 randomized controlled studies. The results reported in the contemporary medical literature showed that although ECTR significantly reduced postoperative hand pain, it increased the possibility of reversible postoperative nerve injury in patients with idiopathic CTS. No statistical difference in the overall complication rate, subjective satisfaction, the time to return to work, postoperative grip and pinch strength, and operative time was observed between the two groups of patients.
